# Autopsy-Confirmed Undifferentiated Carcinoma of the Liver With Rapid Bone Metastasis

**DOI:** 10.7759/cureus.49848

**Published:** 2023-12-02

**Authors:** Keigo Kobayashi, Norikuni Shibata, Kenji Ohmoto, Masako Omori, Yasuhiro Umekawa

**Affiliations:** 1 Department of Internal Medicine, Kurashiki Medical Center, Kurashiki, JPN; 2 Division of Hepatology, Kurashiki Medical Center, Kurashiki, JPN; 3 Division of Pathology, Kurashiki Medical Center, Kurashiki, JPN

**Keywords:** undifferentiated carcinoma of liver, nivolumab, cancers of unknown primary, metastatic iliac bone tumor, autopsy

## Abstract

A male in his late 70s, with progressive hip pain, was diagnosed with a metastatic iliac bone tumor, initially misinterpreted as septic arthritis. Despite extensive imaging, the primary tumor site was undetected until autopsy, which confirmed undifferentiated carcinoma of the liver with secondary lung involvement. The patient's poor performance status and rapid health decline precluded aggressive treatment, leading to his demise. This case underscores the difficulties in diagnosing such carcinomas and suggests that early identification and innovative therapies like nivolumab could potentially extend life in similar cases.

## Introduction

Undifferentiated carcinoma of the liver is a rare malignant neoplasm [1,2]. The clinicopathological features and progression of this carcinoma are not well-documented, and no definitive therapeutic approach exists for inoperable instances [3].

Typically, undifferentiated carcinoma of the liver manifests as liver tumors, frequently accompanied by recurrent fever and inflammatory responses, leading to potential misdiagnosis as hepatic abscesses [2]. Contrarily, our patient exhibited a pronounced metastatic bone tumor in the iliac bone with no identifiable primary site, even upon comprehensive imaging. An autopsy later confirmed it as an undifferentiated hepatocellular carcinoma.

Following the patient's rapid decline in performance status (PS) after hospitalization, initiating aggressive treatment amidst an early diagnosis could have been challenging, casting doubt on the possibility of a positive outcome. By presenting this case, we intend to provide clinicians with a reference for potential analogous situations in the future, with the hope of facilitating treatments in a more favorable PS setting.

## Case presentation

**Figure 1:**
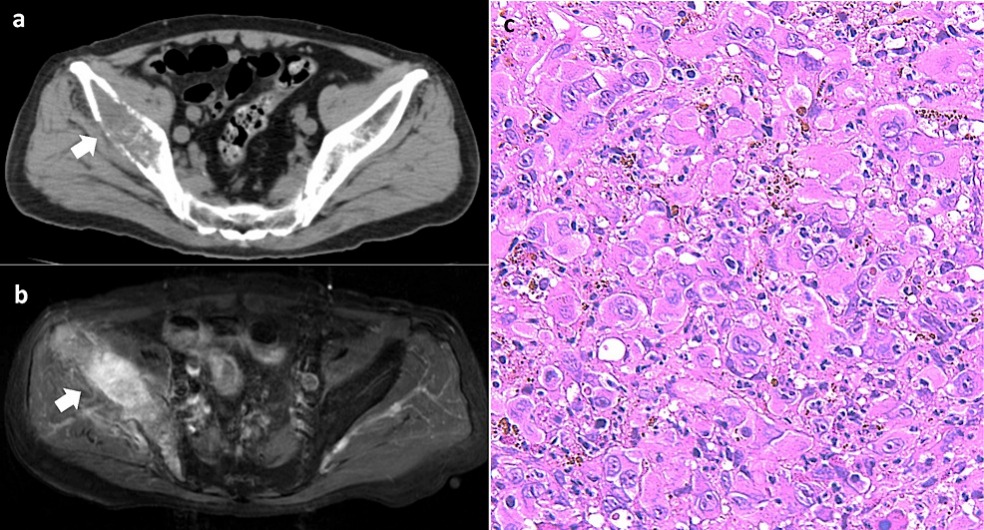
Imaging and pathology of the bone lesion a. Pelvic plain CT: osteolytic changes of the trabecular bone and cortical thinning were observed in the right ilium (white arrow). b. Contrast-enhanced pelvic MRI (fat-suppressed T1-weighted image): the mass in the right ilium showed intense enhancement with the contrast agent (white arrow). c. Histological image of the ilium biopsy (hematoxylin and eosin stained, 400x magnification): The tumor cells with a round morphology exhibited a densely proliferating, poorly differentiated, and highly malignant phenotype.

A male in his late 70s sought medical consultation due to progressive pain in his right hip joint, commencing a month earlier and resulting in walking difficulty. Elevated inflammatory markers (C-reactive protein (CRP) 10.43 mg/dL) from blood tests initially hinted at septic arthritis. However, hip joint aspiration negated this suspicion. CT imaging revealed osteolytic alterations in the right ilium and bone cortex thinning (Figure 1a), prompting concerns of either osteomyelitis or a bone tumor, either primary or metastatic. Yet, the absence of response to antibiotic treatment ruled out osteomyelitis. A subsequent contrast-enhanced MRI of the pelvis unveiled a tumorous lesion in the right ilium (Figure 1b), culminating in a bone tumor diagnosis. To gather more insights, a CT-guided bone biopsy was performed. The histopathology showcased poorly differentiated, high-grade tumor cells with a roundish morphology (Figure 1c), and only CK7 and CK AE1/AE3 tested positive in immunohistochemistry (Table 1). Although osteosarcoma with cytokeratin expression is uncommon, such instances have been documented [4], with significant osteoid formation as a characteristic feature. However, in this patient, the dominance of osteolytic changes, as opposed to the osteoid production seen in typical primary cytokeratin-positive bone tumors, suggested a different pathology. The constellation of these findings, coupled with the immunohistochemical profile, refuted the likelihood of primary bone sarcomas and hematologic malignancies, pointing instead to a diagnosis of metastatic carcinoma to the bone.

**Table 1:** Summary of immunostaining results

	vimentin	CK7	AE1/AE3	CK20	CAM5.2	HepPar1	Arginase	TTF-1	CA19-9	CEA	G-CSF	S-100	α-SMA	CD34	CD10	CD30
Liver	（＋）	（＋）	（－）	（－）	（－）	（－）	（－）	（－）	（－）		（－）					
Lung		（－）		（－）				（－）	（－）							
Bone		（＋）	（＋）	（－）	（－）	（－）	（－）	（－）	（－）	（－）	（－）	（－）	（－）	（－）	partially（＋）	（－）

**Figure 2:**
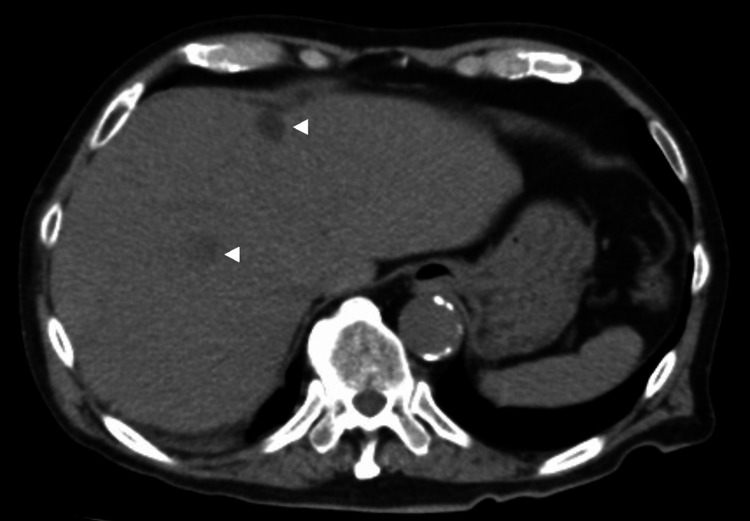
No abnormal liver lesions Abdominal plain CT: No abnormal findings were observed, except for a few hepatic cysts (white triangle).

An exhaustive search to pinpoint the primary tumor site yielded no other tumorous findings, with abdominal CT (Figure 2) and ultrasound revealing just several hepatic cysts and no other anomalies. No detectable tumorous growths in the lungs, biliary system, or kidneys were seen, and significant lymphadenopathy was absent. The patient's liver enzyme levels were within normal ranges, including aspartate aminotransferase (AST) at 34 IU/ml, alanine transaminase (ALT) at 37 IU/ml, and gamma-glutamyl transpeptidase (γGTP) at 59 IU/ml. Additionally, tests for liver infections were negative for both hepatitis B virus (HBV) and hepatitis C virus (HCV). All tumor markers measured were also within normal limits, with prothrombin induced by vitamin K absence-II (PIVKA-II) at 23 mAU/ml, cancer antigen 19-9 (CA19-9) at 2 U/ml, carcinoembryonic antigen (CEA) at 2.7 ng/ml, cytokeratin-19 fragment (CYFRA) at 3.2 ng/ml, sialyl Lewis-x antigen (SLX) at 29 U/ml, and prostate-specific antigen (PSA) at 1.5 ng/ml. Remarkably, the iliac bone tumor underwent swift expansion, underscored by rapid cortical bone destruction. Given the patient's senior age and deteriorating PS, opting for aggressive treatments, including chemotherapy, was deemed inappropriate. His condition was further compromised by complications such as hypercalcemia-induced cognitive disturbances, a hemorrhagic duodenal ulcer, septicemia, and urinary infections. Despite intervention attempts, the patient succumbed on his 84th hospital day.

**Figure 3:**
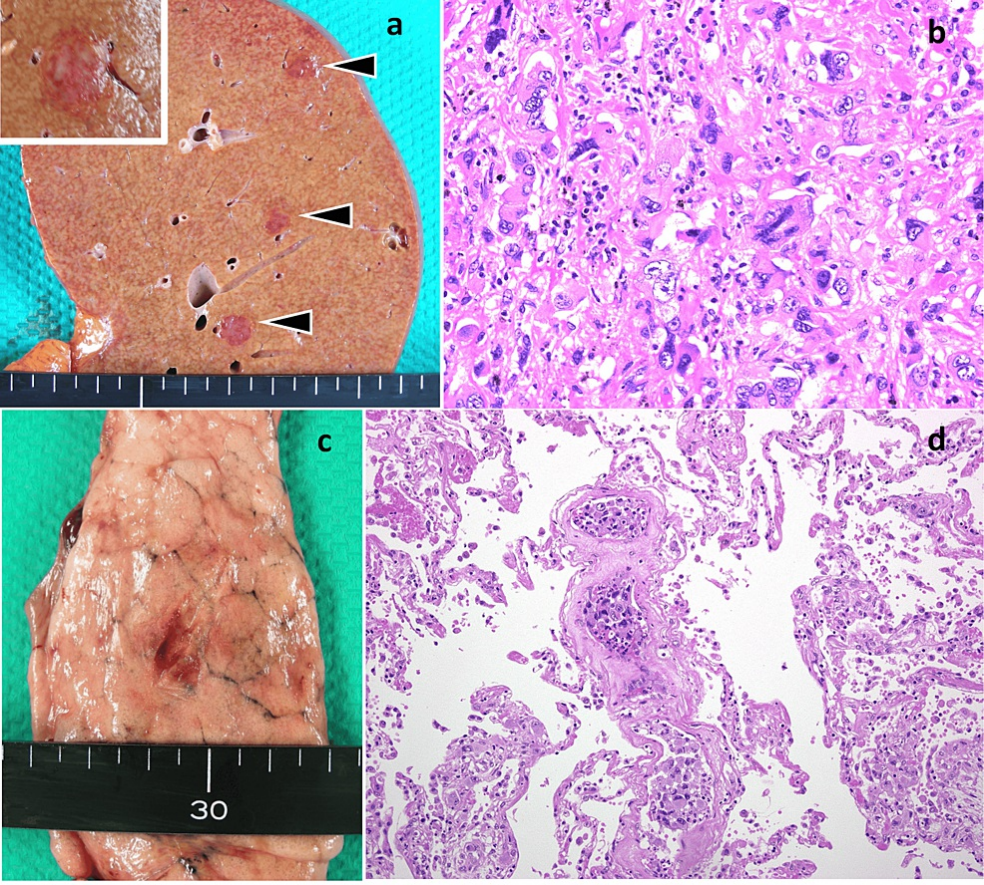
Dissection and histopathology of liver and lung lesions a. Liver dissection: multiple nodular lesions were identified (black triangle). b. Histological image of the liver tumor (hematoxylin and eosin stained, 400x magnification): highly malignant tumor cells with a round morphology were densely proliferating. c. Lung dissection: the overall appearance was mostly normal, but a few palpable nodules were detected. d. Histological image of the lung (hematoxylin and eosin stained, 100x magnification): atypical cells distinct from blood cells were seen throughout the lung, showing significant vascular invasion.

A post-mortem analysis conducted five hours after his passing identified nodular lesions proliferating in the liver (Figure 3a). The histopathological assessment mirrored the bone tumor findings (Figure 3b), with CK7 and vimentin positivity in immunohistochemistry confirming this (Table 1). This, alongside the observed vascular invasion of atypical cells in the lung (Figures 3c, 3d), steered the diagnosis toward undifferentiated carcinoma originating from the liver.

## Discussion

Undifferentiated carcinoma of the liver is an exceedingly rare entity. Primary liver epithelial tumors can develop into two types, hepatocellular and cholangiocellular. However, undifferentiated carcinomas remain in an undeveloped state and proliferate without differentiation, particularly when they are at the point of becoming hepatocellular in nature [5]. Histopathologically, these undifferentiated tumors are marked by densely packed tumor cells with small, round nuclei and lack any distinct features of differentiation [6]. The atypical CK7 positivity detected immunohistochemically in this case, an uncommon trait for hepatocellular carcinoma, combined with vimentin positivity - a marker characteristic of mesenchymal cells - further solidified the diagnosis.

Though tumor nodules were discerned in the patient’s lungs, three key factors underscored the liver as the primary tumor site. First, the lung tumor nodules were markedly fewer and smaller compared to those in the liver. Second, the predominant histological feature of the lung was the vascular invasion orchestrated by anomalous cells. Lastly, the congruence in CK7 immunostaining between the liver and bone tumors further credence to this determination.

In the context of bone metastatic tumors, existing literature posits that lung, breast, thyroid, kidney, and liver cancers are frequent culprits for the primary malignancy. Strikingly, the diagnosis of primary cancer often follows the detection of bone metastasis, which, in many cases, is the initial presenting symptom [7]. This notion is further substantiated by data revealing that nearly 30% of patients manifesting bone metastasis are devoid of a discernible primary tumor upon their initial diagnosis [8,9].

The diagnostic challenge in this case was augmented by the absence of tumorous lesions external to the bone, a departure from the typical presentation of undifferentiated carcinoma of the liver. This anomaly underscores the conundrum surrounding the therapeutic approach.

While undifferentiated carcinoma of the liver lacks a definitive treatment protocol [1-3], nivolumab, an immuno-checkpoint inhibitor, has shown promise for those cases classified under the 'poor prognosis group' of cancers of unknown primary [10]. These elusive cancers are bifurcated clinically and pathologically into two main categories. The 'better prognosis group', constituting 15-20%, can be approached with curative intent while the 'poor prognosis group', making up 80-85%, has no standardized treatment regimen [11]. For patients in the latter category, should their PS permit, nivolumab administration might proffer a notable extension in overall survival compared to traditional chemotherapy. This becomes evident when contrasting the historical median overall survival of eight months with empirical chemotherapy to a significantly improved 16.2 months using nivolumab [10,12].

Regrettably, in the presented case, the patient's initial PS of 2 swiftly regressed to 4 within a mere month of hospitalization. Given the advanced age of the patient, aggressive therapeutic interventions could have potentially been detrimental to the overall prognosis.

## Conclusions

The overarching message this case imparts is the imperative for swift and comprehensive evaluations in the backdrop of rapidly advancing bone metastatic tumors. A timely diagnosis, especially when the patient’s PS remains favorable in the 'poor prognosis group' of cancers of unknown primary, can pave the way for potentially life-extending treatments such as nivolumab. This case serves as a reminder of the unyielding effort needed for a swift and accurate cancer diagnosis, particularly when dealing with a potential cancer of unknown primary.
